# *In vivo* label-free structural and biochemical imaging of coronary arteries using an integrated ultrasound and multispectral fluorescence lifetime catheter system

**DOI:** 10.1038/s41598-017-08056-0

**Published:** 2017-08-21

**Authors:** Julien Bec, Jennifer E. Phipps, Dimitris Gorpas, Dinglong Ma, Hussain Fatakdawala, Kenneth B. Margulies, Jeffrey A. Southard, Laura Marcu

**Affiliations:** 1 Department of Biomedical Engineering, University of California Davis, Davis, 95616 CA USA; 2 Cardiovascular Institute, Perelman School of Medicine, University of Pennsylvania, Philadelphia, 19104 PA USA; 3 UC Davis Health System, Division of Cardiovascular Medicine, University of California Davis, Sacramento, 95817 CA USA; 4 Institute of Biological and Medical Imaging, Present Address: Helmholtz Zentrum, München, Germany; 5 Present Address: Abbott, Sylmar, CA USA

## Abstract

Existing clinical intravascular imaging modalities are not capable of accurate detection of critical plaque pathophysiology in the coronary arteries. This study reports the first intravascular catheter combining intravascular ultrasound (IVUS) with multispectral fluorescence lifetime imaging (FLIm) that enables label-free simultaneous assessment of morphological and biochemical features of coronary vessels *in vivo*. A 3.7 Fr catheter with a fiber-optic channel was constructed based on a 40 MHz clinical IVUS catheter. The ability to safely acquire co-registered FLIm-IVUS data *in vivo* using Dextran40 solution flushing was demonstrated in swine coronary arteries. FLIm parameters from the arterial wall were consistent with the emission of fluorophores present in healthy arterial wall (collagen, elastin). Additionally, structural and biochemical features from atherosclerotic lesions were acquired in *ex vivo* human coronary samples and corroborated with histological findings. Current results show that FLIm parameters linked to the amount of structural proteins (e.g. collagen, elastin) and lipids (e.g. foam cells, extracellular lipids) in the first 200 μm of the intima provide important biochemical information that can supplement IVUS data for a comprehensive assessment of plaques pathophysiology. The unique FLIm-IVUS system evaluated here has the potential to provide a comprehensive insight into atherosclerotic lesion formation, diagnostics and response to therapy.

## Introduction

Myocardial infarction due to plaque rupture is a leading cause of sudden cardiac death^1^. Due to positive remodeling, high plaque burden may occur without significant stenosis, and therefore cannot be reliably identified using conventional angiography. Intravascular ultrasound (IVUS) enables identification of plaque burden due to its high penetration depth (up to 10 mm^2^) but lacks the spatial resolution to identify small scale features such as details of the intima^3^, or biochemical changes linked with atherosclerotic lesion formation and evolution. Optical coherence tomography (OCT) provides improved spatial resolution (<20 µm^2^) that enables highly detailed visualization of fibrous cap and intima morphology as well as some level of detection of macrophages^4^ at the cost of penetration depth, thus limiting the ability to assess plaque burden. Direct access to biochemical information could prove valuable for the study of atherosclerotic lesion pathophysiology. Previous studies showed that spectroscopic techniques including near infrared (NIR)^5, 6^, Raman^7^ and fluorescence^8, 9^ have the potential to provide biochemical information. Thus there is a growing interest in developing “orthogonal” multimodal techniques combining biochemical and structural imaging capabilities^10, 11^, which is the focus of this study.

Previous studies have demonstrated that fluorescence lifetime techniques based on UV- light induced tissue autofluorescence^12, 13^ can complement IVUS to better identify distinct coronary plaque pathologies. In previous work, we developed a catheter with a small cross-section profile (3.2 Fr) relying on sequential scanning of the field of view by independent FLIm and IVUS imaging cores integrated into a single sheath and imaging section^14^. The catheter was then used to interrogate *ex vivo* human coronary arteries. The study showed that this label-free technique combining IVUS and FLIm can differentiate between 8 different plaque subtypes, identifying for example thin cap fibroatheroma (TCFA) with 90% sensitivity and 100% specificity using pathological features such as the presence of macrophages in the fibrous cap. Thus, this bi-modal technique is suitable for identification of plaque phenotypes known to be responsible for acute coronary syndromes. However, the embodiment of the FLIm-IVUS catheter technology used in this previous work was not suitable for *in vivo* use, as the device was not flexible enough and the sequential scanning scheme limited the ability to co-register data in the presence of motion. Additionally, *in vivo* coronary imaging requires displacement of blood with an optically transparent solution.

We demonstrate here how we successfully addressed these challenges by integrating IVUS and FLIm elements into a compact, single rotational intravascular catheter (3.7 Fr) able to simultaneously acquire co-registered structural (via IVUS) and biochemical (via FLIm) images of coronary arteries *in vivo* in a single pull-back. The technical performance of the bi-modal system assessed with artery phantoms. The ability of the system to acquire robust bi-modal data in coronary arteries *in vivo* using standard percutaneous coronary intervention techniques in combination with a Dextran solution bolus flush was demonstrated in healthy swine. Imaging of a few representative diseased human samples was performed and showed that different types of lesions in diseased coronary arteries, identified via histology, are characterized by FLIm biochemical signatures consistent with findings from earlier studies.

New imaging techniques for evaluation of plaque pathophysiology are of great interest to both improve the understanding of mechanisms driving plaque formation as well as support the development of new preventative, pharmaceutical and interventional therapies^10^. By providing this information, the presented device could become a valuable addition to the field of cardiovascular imaging.

## Results

### FLIm-IVUS imaging catheter system

**Figure 1:**
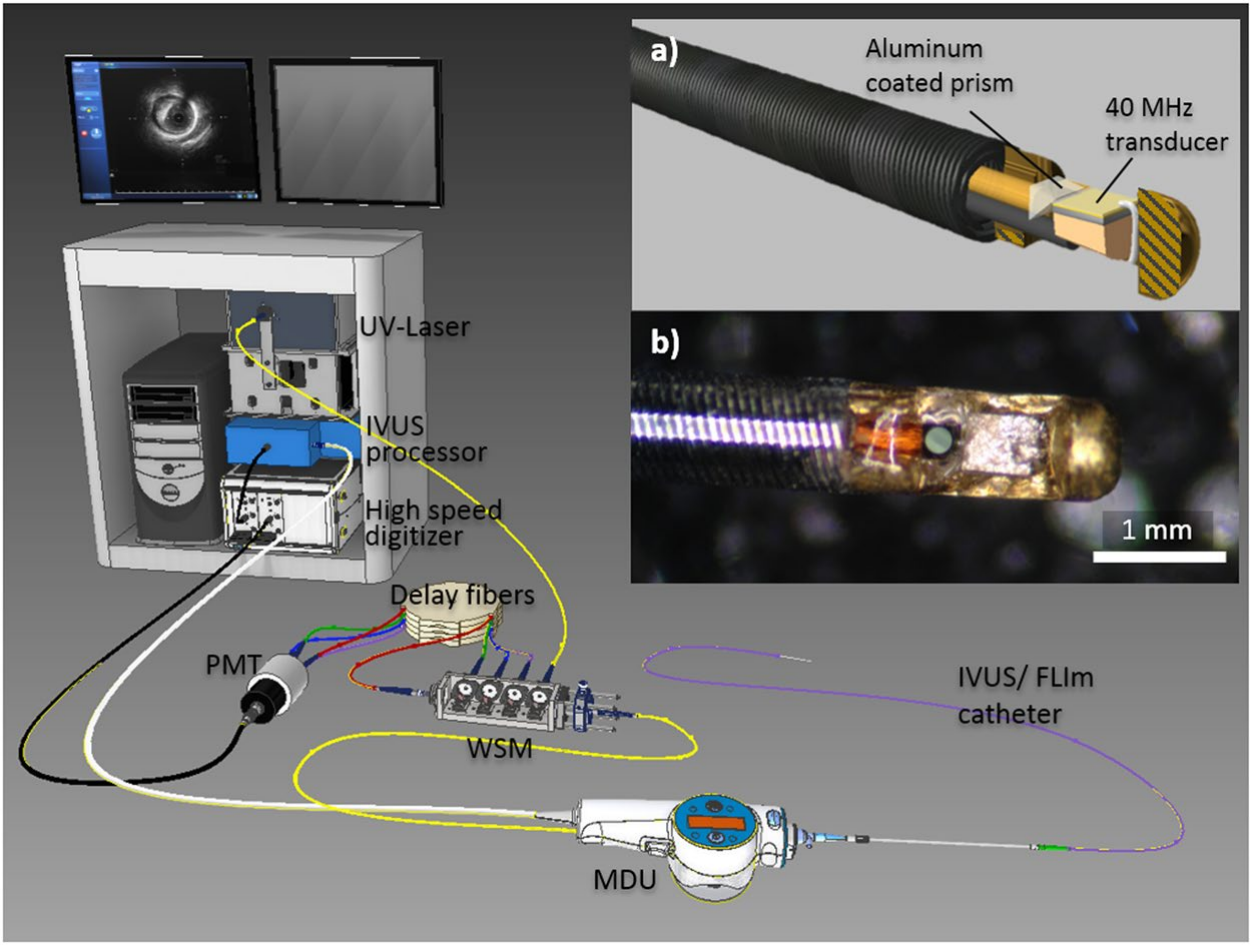
System overview. The system, able to display IVUS images in real time using the clinical interface, is composed of a 3.7 Fr IVUS/FLIm catheter, an IVUS motor drive unit (MDU) modified to accommodate an optical channel, connected to a wavelength selection module (WSM). The WSM enables both coupling of the pulsed excitation light and spectral decomposition of the collected fluorescence signal. The signal from each band is sent to the photomultiplier tube (PMT) using different lengths of optical delay lines that enables measurement over four wavebands using a single detector. When acquiring bimodal data, co-registration is insured by sampling both FLIm and IVUS signal with a single digitizer.

The bi-modal imaging system was developed based on a Boston Scientific iLab™ IVUS system and is composed of a custom IVUS/FLIm rotational catheter, a modified Boston Scientific motor drive unit (MDU), and data acquisition and display units of both IVUS and FLIm sub-systems (Fig. 1).

The size (3.7 Fr) of the FLIm/IVUS catheter reported here is compatible with coronary imaging. In its current configuration, the IVUS performance (central frequency and bandwidth) is similar to the standard 40 MHz OptiCross catheter. The modified MDU provided a robust interface with the catheter, with optical coupling losses of less than 1 dB through the MDU.

The system enables scans of a 20 mm section of vessel in 5 seconds with a rotation speed of 1800 rpm, providing 25000 independent multispectral fluorescence lifetime point measurements of the vessel surface (20 kHz laser repetition rate, 4 time average per point), co-registered with IVUS data. The data analysis software allows for display of the intensity *en face* images derived from FLIm within seconds following the scan and the fast data processing based on Laguerre technique enables the computation and display of lifetime maps from all 4 spectral channels in less than 2 minutes following the end of the scan.

### *In vivo* FLIm/IVUS imaging of healthy coronary arteries

The ability of the bi-modal imaging system to access coronary arteries, flush the blood from the field of view and acquire robust autofluorescence data *in vivo* was evaluated in two swines. The characterization was focused on the following: (i) ability of the catheter to access tortuous anatomy, (ii) ability to flush blood from the field of view, (iii) ability to image a range of vessel diameters, and (iv) ability to discriminate targets based on lifetime and spectral information.

**Figure 2:**
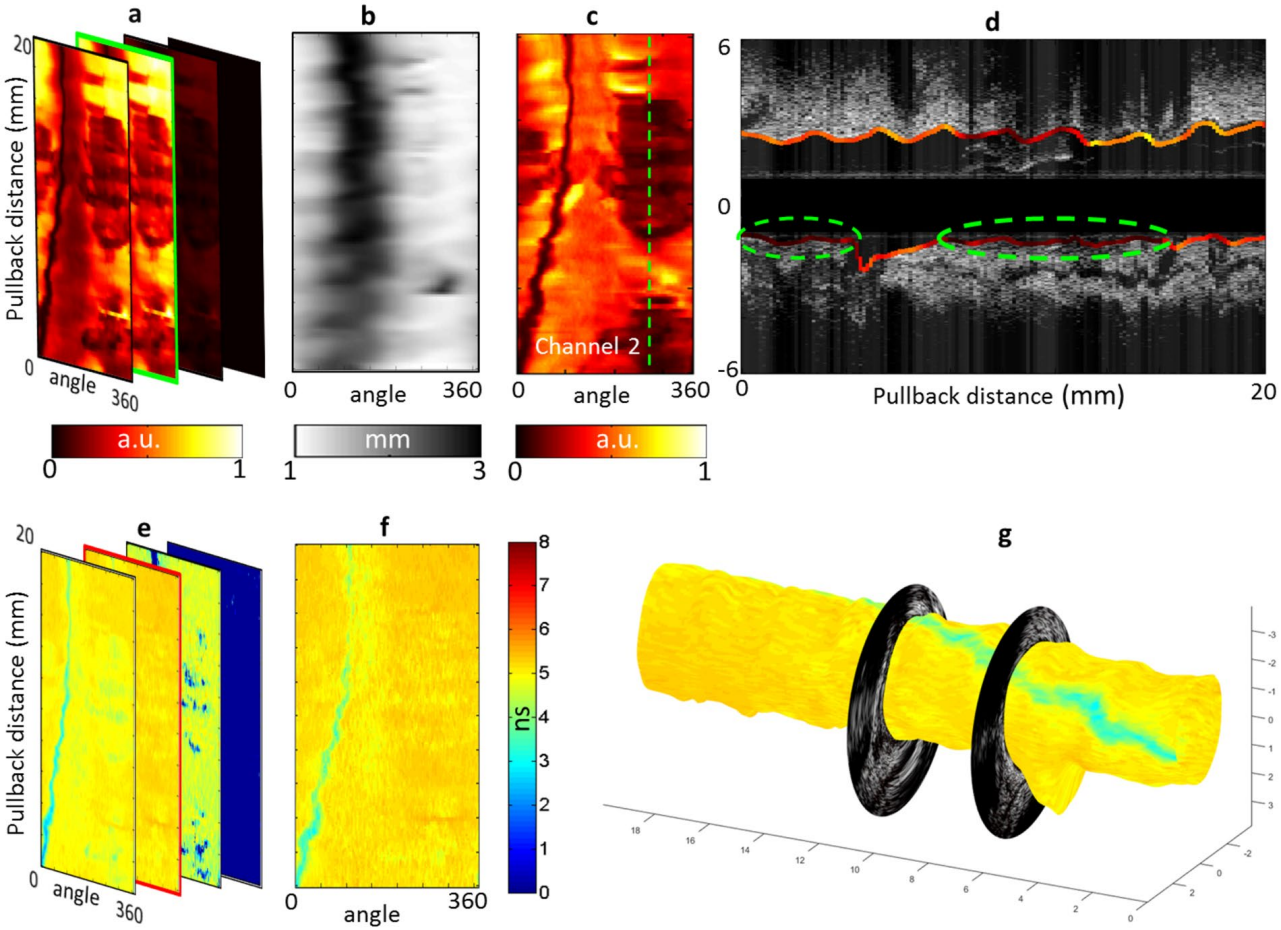
Ability to acquire co-registered FLIm/IVUS data *in vivo* in coronary arteries was evaluated in swine. Intensity *en face* image of a section of left circumflex artery acquired *in vivo* in pig (**a**). A distance map derived from IVUS (**b**) enables distance correction of the intensity image (**c**). For each of the four channels, a lifetime image is derived (**e**). Channel 2 presents the most fluorescence due to presence of collagen and elastin and shows very uniform background as expected from a healthy pig (**f**). The FLIm data can be combined with IVUS to provide bimodal 3D renderings (**g**) as well as sections of the vessel (**d**) (30° orientation, corresponds to the dashed line of panel c).

Swine data obtained from a co-registered FLIm/IVUS acquisition performed *in vivo* in a 3 mm diameter section of the circumflex artery using a 5 cc/s flushing rate are presented in Fig. 2. Strong fluorescence emission was recorded in channels 1 and 2 (Fig. 2a), whereas emission is low in channel 3 and absent in channel 4, matching the emission of collagen and elastin (see supplemental material). A map of the probe-to-vessel wall distance (Fig. 2b) was extracted from IVUS data and used to correct for the effect of probe-to-wall distance on the intensity data (Fig. 2c). We observe the guidewire shadow as well as low intensity areas centered on the green dotted line shown in Fig. 2c. A longitudinal section across this line (Fig. 2d) shows that the catheter is in contact with the wall in these areas. In this configuration, blood can be trapped between the device and the vessel wall: the increased absorption of light from blood in both excitation and autofluorescence emission wavelengths creates these low intensity areas.

Fluorescence lifetime is an inherently ratiometric technique and presents the ability to extract lifetime values independent from intensity for all but the lowest measured fluorescence signal (typically SNR > 15 dB, as reported previously^15^). This property is verified by computing lifetime maps retrieved from each channel (Fig. 2e,f). After excluding the guidewire location, we verified that recovered lifetime values were uniform across the field of view as expected from a healthy artery. The recovered values for this 3 mm diameter vessel were as follows 4.98 ± 0.20 ns (channel 1), 5.19 ± 0.12 ns (channel 2), 4.55 ± 0.29 ns (channel 3), and no significant emission in channel 4. This was achieved in spite of wide variations of intensity over the field of view and noticeable signal absorption by blood in some areas, as described above. The independence of the lifetime information from signal intensity is further presented in the supplemental material. 3D rendering of the fluorescence lifetime image was also created to enable easy co-location of FLIm and IVUS features (Fig. 2g).

**Figure 3:**
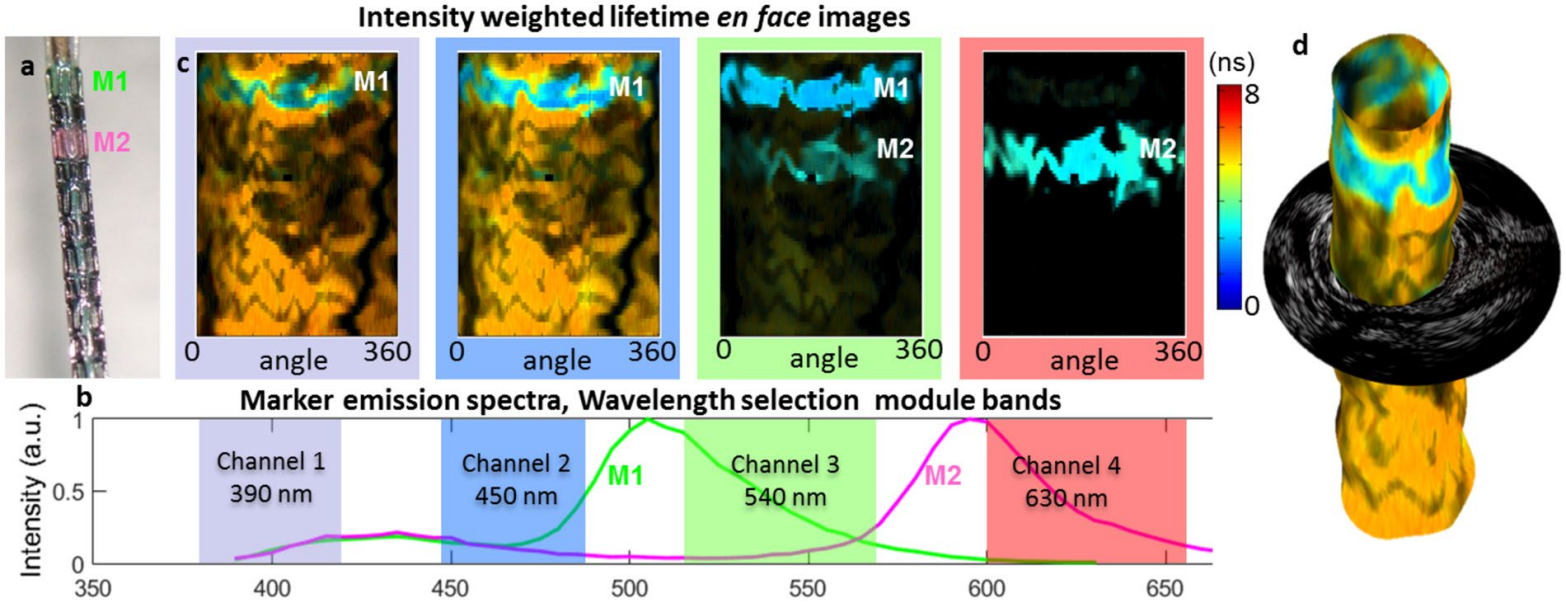
The ability of FLIm to resolve targets using fluorescence lifetime and spectral contrast was evaluated *in vivo* in swine coronary arteries. *In vivo* images of stented section of swine circumflex artery. Fluorescent markers M1 & M2 are painted onto the stent to provide fluorescence contrast (**a**), present broad emission spectra (**b**). M1 emission presents a short (~3 ns) lifetime and can be easily discriminated from healthy vessel background (~5.3 ns) in channels 1 and 2. M2 has weaker emission in channels 1 and 2 but is readily identified in channel 4 (**c**). The ability to resolve fluorescent features based on both spectral and temporal parameters makes FLIm a powerful technique to discriminate areas of interest. Combining FLIm and IVUS images enables 3D rendering (**d**).

The ability of the catheter system to differentiate targets based on FLIm information (lifetime, spectra) was evaluated by acquiring multimodal data in a stented section as presented in Fig. 3. Fluorescence targets were painted on the stent (Fig. 3a) to provide contrast with respect to the vessel wall autofluorescence. Intensity weighted lifetime maps, where the color represents the lifetime value and the brightness represents the amount of signal, are displayed for each channel (Fig. 3c). As presented in the normalized emission spectra of both markers (Fig. 3b), the marker M1 emission is overlapping with the vessel autofluorescence emission in channels 1 and 2, but can be readily identified in these channels due to its shorter lifetime (~3.3 ns). Additional discrimination is provided by the spectral information: the peak emissions of markers M1 and M2 in channels 3 and 4 respectively provide clear identification of the location of the markers. The 3D rendering (Fig. 3d) obtained by combining FLIm and IVUS information illustrates how areas of interest identified using FLIm data can be localized within the vessel morphology representation.

Imaging large vessels presents challenges for fluorescence signal acquisition as the excitation collection efficiency decreases with probe-to-wall distance. When imaging a 5 mm diameter section of vessel, the collected fluorescence signal in the region of interest farthest from the probe was reduced by 14.5 dB with respect to the area reported for the 3 mm vessel. For this second region, the recovered lifetime values (e. g. 5.14 ± 0.33 ns in channel 2) were comparable with those obtained for smaller vessels (5.19 ± 0.12 ns in channel 2), and ensure that the system can collect data for all vessel diameters expected during coronary intervention. To evaluate the flushing parameters, the flushing flow was varied between 2 cc/s and 8 cc/s. While flushing flow below 4 cc/s did not lead to proper clearing of the blood from the field of view, no noticeable difference in flushing efficiency were identified for both smaller and larger vessel between 4, 6 and 8 cc/s flushing rates.

### *Ex vivo* plaque characterization using FLIm and IVUS data

**Figure 4:**
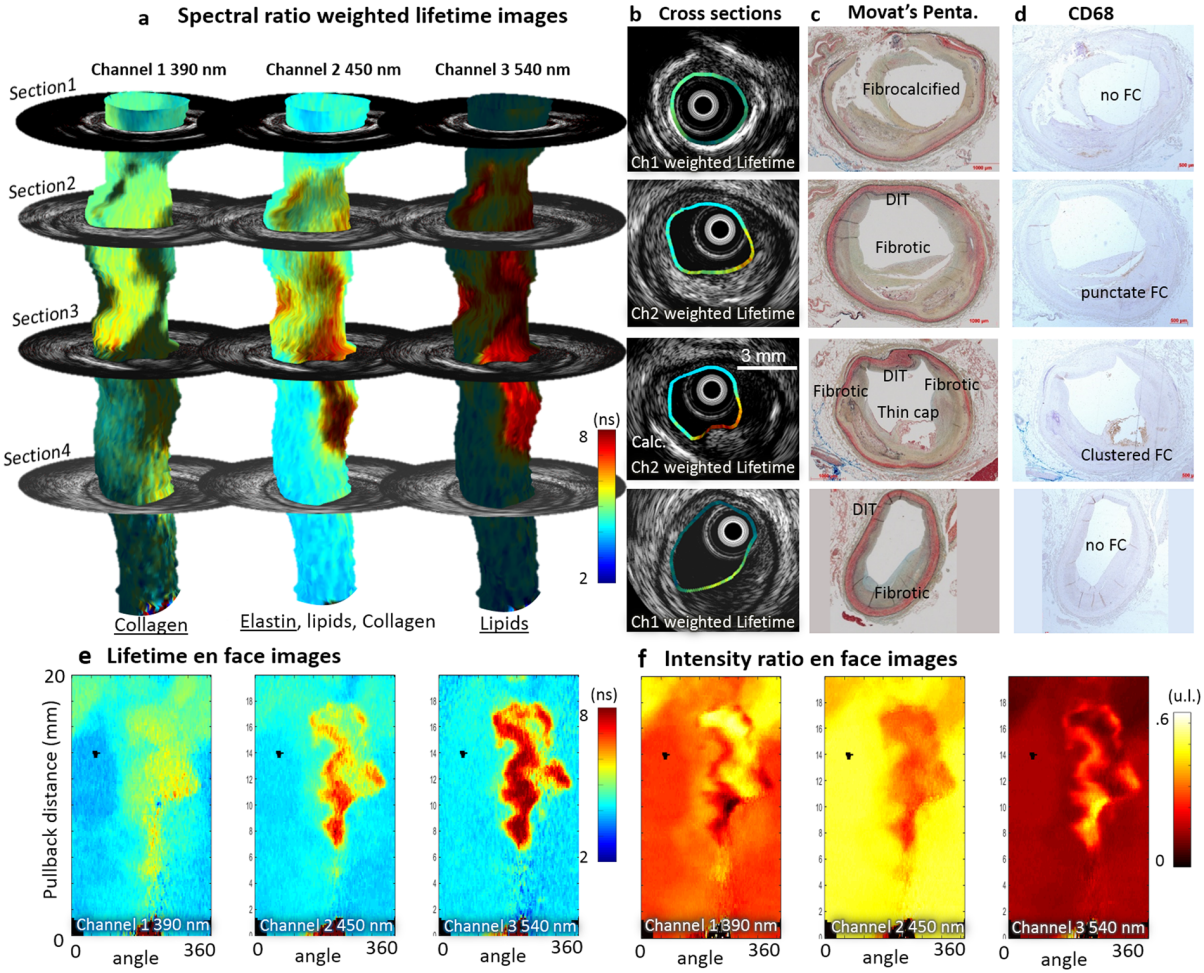
FLIm biochemical information supplements IVUS for the assessment of atherosclerotic lesion pathophysiology. Spectral ratio weighted lifetime images map the measured lifetime (color), as well as relative strength of the signal (brightness) for channels 1 (Collagen), 2 (Elastin, some collagen and lipids) and 3 (lipids, ceroid and lipofuscine) of the instrument. Section 1 presents a fibrocalcified lesion. Areas of superficial calcification (3 to 9 o’clock) present less fluorescence from collagen (channel 1) than the rest of the vessel. Section 2 presents diffuse intimal thickening (DIT, upper quadrant) as well as a fibrotic lesion with a deeper necrotic core (lower quadrant), easily identifiable in the IVUS cross section. The increased presence of collagen in the fibrotic area corresponds to high fluorescence intensity in channel 1. Infiltration of punctate foam cells (FC) in the lower right quadrant (see CD68) corresponds to increased lifetime in channel 2 and some low level fluorescence in channel 3. Section 3 presents a thin cap fibroatheroma (TCFA) in the lower right quadrant. The fluorescence signature of this area is characterized by an absence of channel 1 signal (no collagen) and long lifetime in channels 2 and 3 with a maximum emission in channel 3 (large amounts of lipids/ceroid/lipofuscine). Other locations in the section present a signature expected from DIT/fibrotic lesions (collagen/elastin). Calcification from 6–9 o’clock is readily detected by IVUS. Section 4 presents DIT (top, left) and a fibrotic lesion (bottom, right). The fibrotic area can be differentiated from DIT by its higher collagen content (increased channel 1 intensity, longer channel 2 lifetime). The absence of infiltrated FC (see CD68) in this fibrotic area is consistent with the much lower lifetime observed here with respect to the fibrotic region in section 1. Proteoglycans, identified by the alcian blue color in the 6 to 8 o’clock region of the Movat’s section may explain the increase in lifetime observed at this location in the channel 1 image. En face images of lifetime (**e**) and intensity ratio (**f**) used to compute ratio weighted lifetime images shown in (**a**).

We present results from two representative cases chosen from human coronary arteries imaged *ex vivo* to illustrate the typical contrast provided by FLIm in diseased vessels. We will also use this data to illustrate how FLIm-derived compositional features can complement the IVUS-derived morphological features (e.g. lumen geometry, plaque thickness and presence of calcifications) enabling a more comprehensive evaluation of plaque pathophysiology, The cases consist of datasets of co-registered IVUS and multispectral FLIm from 20 mm-long arterial segments, with matching histology sections. The use of a dedicated sample holder during imaging enables the accurate co-registration of the imaging data with histology sections. One example is presented in Fig. 4. Results from a second artery are presented in the supplemental materials (Figs s6 and s7).

### Ability of FLIm to resolve distinct cap compositions

The FLIm data are represented using spectral ratio-weighted lifetime maps, projected onto the vessel lumen segmented from the IVUS data (Fig. 4a). This representation enables both time-resolved and spectral information to be included in the same graphic representation: the lifetime measured in each of the four channels of the instrument is color-coded (see Fig. 4e), whereas each pixel’s brightness is weighted by the share of the total measured fluorescence intensity originating from this specific channel, or intensity ratio, represented in Fig. 4f (See supplemental methods section for additional details). These maps are independent from variations in excitation/collection efficiency due to distance and geometry and reflect intrinsic fluorescence properties of the superficial layer of the arterial wall (~200–300 µm^16^). These data are then compared with histology sections stained with Movat’s pentachrome (Fig. 4c) and CD68 (Fig. 4d) obtained from four different axial locations.

Section 1 presents a fibrocalcified lesion. The upper region (9 to 3 o’clock) presents strong signal from collagen, as expected from a fibrous lesion. Calcified areas at the luminal surface (3 to 9 o’clock), easily identified from IVUS data and confirmed on H&E section (not shown) are characterized by a low fluorescence contribution from collagen (channel 1). Section 2 presents diffuse intimal thickening (DIT) in the upper quadrant as well as a fibrotic lesion with a deeper necrotic core (lower quadrant), easily identifiable in the IVUS cross section. The strong presence of collagen in the fibrotic area matches the high fluorescence intensity in channel 1. Infiltration of punctate foam cells (FC) in the lower right quadrant (See Fig. 4d, CD68) corresponds to increased lifetime in channel 2 ( > 5.5 ns) and presence of some fluorescence in channel 3. Section 3 presents a clear instance of thin cap fibroatheroma (TCFA) in the lower right quadrant. The fluorescence signature of this area is characterized by an absence of channel 1 signal (low collagen content) and long lifetime in channels 2 (>6.5 ns) and 3 (>7.5 ns) with a maximum emission in channel 3. This large increase of lifetime may be linked to a change in the lipid composition observed in advanced lesions^17–19^. Other locations in the section present a signature expected from DIT/fibrotic lesions (collagen/elastin). Calcification from 6–9 o’clock is readily detected by IVUS. Section 4 presents DIT (top, left) and a fibrotic lesion (bottom, right). The fibrotic area can be differentiated from DIT by its higher collagen content (increased channel 1 intensity, longer channel 2 lifetime). In this fibrotic location, no infiltrated FC are visible (see Fig. 4d, CD68). This is consistent with the lifetime signature, characteristic of collagen/elastin emission and much lower than values from the fibrotic region in section 2 where foam cell infiltration was observed. The presence of proteoglycans is identified by the alcian blue color in the 6 to 8 o’clock region of the Movat’s section. Reported fluorescence properties^20^ are consistent with the increase in lifetime and intensity observed at this location in the channel 1 image.

The ability of FLIm to provide contrast based on both spectral and temporal information enables the delineation of various areas based on FLIm signature. While the exact source of molecular contrast is difficult to confirm with certainty (emission spectra of various compounds overlap and may contribute to the overall fluorescence signatures) the areas delineated by FLIm match well with specific plaque compositional features identified from histology.

### Ability to resolve morphology

IVUS is well known for its ability to resolve vessel morphology, including lumen shape and plaque burden, due to its large penetration depth. As expected, the overall vessel shape (lumen geometry, intima thickness) observed from the acquired IVUS B-scans is very similar to the vessel geometry identified in histology sections of the corresponding axial locations; this provides a convenient way to independently verify the accuracy of imaging/histology registration. Additionally, the presence of calcification is easily identified in both IVUS and Movat’s pentachrome sections (see Fig. 4, 2^nd^ section, lower left quadrant) and also provides a clear landmark that can be used to further assess registration.

## Discussion

This study demonstrates for the first time that IVUS and FLIm imaging techniques can be integrated into a single catheter to acquire co-registered structural and biochemical information *in vivo* in swine coronary arteries, without the need for exogenous contrast agents. Integration of both imaging modalities was achieved so that the performance of the clinical IVUS component was not negatively impacted by the addition of FLIm capability. More specifically, the current catheter design could access coronary arteries using standard percutaneous coronary intervention techniques in conditions representative of clinical use. We note that while in its current configuration, the image acquisition speed of the FLIm-IVUS system is limited by the framerate of the clinical IVUS from which it derives, in turn limiting the pullback length for a 5 s bolus flush to 20 mm, the main limitation of the current system The FLIm instrumentation employed enables point measurements frequencies of up to 100 KHz, corresponding to 150 frames per second (fps), compared to 30 fps of the clinical IVUS. This constraint is shared with combined IVUS-OCT and is the object of further investigation^21^. A general trend towards increased framerate for the next generation of clinical IVUS^22^ will be leveraged to build a faster FLIm/IVUS system.

Use of Dextran 40 solution to flush blood from the field of view enabled the collection of bi-modality data. Low molecular weight dextran solution has high optical transparency for UV light and is a cost efficient alternative to iodinated contrast^23, 24^ for intravascular OCT imaging. The flushing parameters (typically 4–5 cc/s and less than 40 mL per sequence) are compatible with clinical use. We demonstrated that the FLIm signal acquired in these experimental conditions is robust and enables differentiation of fluorescent features based on both emission spectra and lifetime differences, for vessels up to 5 mm in diameter, therefore covering the whole range of vessel lumen size expected in patients. The standard deviation of the recovered lifetime (0.1–0.3 ns range) is much lower than the fluorescence contrast observed between different lesions present in human samples (1–4 ns). We posit that the proposed device should therefore have the ability to discriminate plaque features *in vivo*.

The combination of FLIm with a morphological imaging modality such as IVUS is important, because FLIm alone cannot provide morphometric information about lumen size, geometry or direct measurement of lesion size. When projected onto the vessel lumen boundary identified by IVUS, the dimensions of FLIm features can be precisely assessed.

Data was acquired from a limited number of *ex vivo* diseased human arteries. The fluorescence signature of various lesions is consistent with findings from earlier studies from our group^12, 13^. Based on this information, we showed that FLIm complements the well-documented ability of IVUS to identify lumen size, plaque burden and calcification. FLIm provides biochemical details about the composition of the vessel wall and allows straightforward identification of fibrotic plaque or ThCFA through the detection of an increase in collagen and a decrease of elastin content in the superficial layer of the intima^25^. TCFA, characterized by a decrease of elastin and collagen content and the presence of lipids and lipoproteins in the superficial layers of the intima could also be identified using changes in fluorescence signature, distinct from both diffuse intimal thickening and fibrotic plaques. The signal detected in channel 3 did not correspond to any of the pure compounds tested independently (supplemental materials, Fig. s4) but suggests the presence of fluorophores such as ceroid^26^ and lipofuscin^27^, which both have emission spectra consistent with the measured signals and are linked with the activity of inflammatory cells. Additionally, the contribution of proteoglycans to fibrotic plaque autofluorescence may be identifiable. These preliminary findings are very encouraging to support the claim that multispectral FLIm based on UV excitation of tissue autofluorescence is well suited to the intravascular identification of various biochemical species relevant to the study of plaque pathophysiology. In summary, the fluorescence signature observed for different plaque subtypes (across all 20 samples imaged in this study) was in good agreement with fluorescence properties of biochemical/morphological features, as confirmed by histology. Current results support further interrogation of a large number of coronary samples and studies designed to demonstrate statistical differences between distinct subtypes.

Although the proposed method does not provide the depth of the identified biochemical species, the penetration depth of the UV light used for excitation (~200 µm^16^) provides a sectioning effect by limiting the interrogated volume to the superficial layer, known to be of particular relevance to plaque characterization^3^.

Vascular imaging can be performed using both invasive (catheter based) and non-invasive techniques. Non-invasive methods provide obvious benefits by preventing the risks associated with PCI and potentially enabling screening of patients. Such modalities now go beyond angiography and include magnetic resonance angiography (MRA), Coronary Computed Tomographic Angiography (CCTA) as well as functional imaging modalities such as positron emission tomography (PET) and single photon emission computed tomography (SPECT) supported by the development of molecular probes targeting inflammation, hypoxia or calcifications^28^. The ability of CCTA and MRA to evaluate calcifications^29^ and plaque thickness^30^ in coronary arteries has been documented but non-invasive imaging of atherosclerosis in coronary arteries is hampered by radiation exposure for PET/SPECT and CT, limited spatial resolution and image quality as well as motion artifacts^28^ and will require significant technological improvements before potentially replacing intravascular imaging methods. In contrast to CCTA or MRA, IVUS has been able for 20 years to provide clinicians with detailed morphological information about coronary plaque but stenosis and plaque burden alone are poor predictors of acute coronary syndrome^31^. In spite of improvement in spatial resolution provided by OCT in comparison to IVUS, it is also unlikely that morphological information alone will enable identification of rupture-prone plaque^32^. Catheter-based near infrared spectroscopy (NIRS)-ultrasound, now available for clinical use, provides clinicians with “chemograms” that display the presence of lipid in the vessel wall. However, the absence of depth resolution, combined with a relatively high penetration depth (multiple millimeters), may lead to ambiguous information with respect to volume or localization of these lipids, thus limiting the clinical value of the information. Molecular imaging methods such as near infrared fluorescence (NIRF), implemented at the research level in NIRF-OCT multimodal approaches aim to identify lipid-rich and inflamed plaque using both exogenous markers^33, 34^ and label free approaches^35^. The ability of NIRF-OCT to detect inflammation has promising potential. Nonetheless, these modalities lack the ability to assess the presence and potential degradation of structural proteins such as collagen and elastin. In contrast, the bi-modal FLIm-IVUS system reported here is able to evaluate and characterize plaque based on the presence of structural proteins such as collagen and elastin as well as peroxidized lipid-protein complex (ceroid) and foam cells near the surface of the lumen. Combined with the well documented ability of IVUS to evaluate lumen shape, plaque burden and calcification, this instrument provides an exciting option for the *in vivo* study and detection of various plaque subtypes.

This new approach may benefit both patients and researchers alike. Enabling *in vivo* evaluation of plaque type may improve understanding of plaque physiopathology and mechanisms of disruption. In addition, the effect of therapeutic agents on biochemical and structural composition of plaque could be evaluated, leading to a non-invasive method to monitor efforts aimed at acute coronary syndromes prevention.

## Methods

### Integrated FLIm/IVUS system

FLIm is based on a fast pulse sampling technique previously developed by our group^36^. Pulses of UV-light (355 nm, 20 kHz) are sent to the sample and generate fluorescence emission from the arterial wall that results from the autofluorescence of structural proteins (collagen, elastin), as well as lipids and lipoproteins. The UV pulses were delivered to the arterial wall using a fiber optic. The generated autofluorescence signal was collected using the same fiber and spectrally resolved in four distinct wavelength bands: Channel 1 (390 nm), Channel 2 (450 nm), Channel 3 (540 nm) and Channel 4 (630 nm). The correspondence between channels and key fluorophores is presented in Table 1 of the supplemental methods. FLIm imaging capability was integrated into the existing IVUS system by modifying the IVUS MDU to accommodate an optical channel and developing a custom 3.7 Fr monorail bi-modal imaging catheter (Fig. 1). The system is able to display IVUS information in real time using the standard user interface and provides guidance to identify areas of interest, as well as co-registered FLIm/IVUS images as demonstrated in phantom (see supplemental material).

Data were processed to make optimal use of the ability of the system to differentiate biochemical compounds using fluorescence decay characteristics as well as spectral intensities: for each channel, fluorescence lifetime and intensity ratio maps were created. Additional information about data processing is available in the supplemental methods.

### *In vivo* coronary imaging in swine

This study was conducted in accordance with the Guide for the Care and Use of Laboratory Animals^37^. The Institutional Animal Care and Use Committee at the University of California, Davis approved the study for the use of Yorkshire cross swine. Animal care and use was performed by qualified individuals and supervised by veterinarians. All facilities and transportation complied with current legal requirements and guidelines. Anesthesia was used in all surgical interventions. Our animal facilities meet the standards of the American Association for Accreditation of Laboratory Animal Care.

The pigs, in the 60–70 kg range, were subject to general anesthesia and the bi-modal catheter device was introduced into the left circumflex coronary under fluoroscopy guidance through a standard 7 French guiding catheter over a 0.014 inch guidewire. Detail of the protocol can be found in the supplemental materials.

In the first animal, an initial set of bi-modal pull-back scans (n = 10) was acquired in vessel diameters ranging from 1.5 mm to 3.5 mm using non-occlusive flush (4 to 8 cc/s for 10 s) of low molecular weight Dextran solution (10% LMD in 5% Dextrose, Hospira, IL) to remove blood from the field of view. The 2.75 mm stent (Promus, Boston Scientific, MA) with fluorescent markers (Scribbles, Duncan, Fresno, CA) was then placed in the circumflex coronary artery. The stented area, where lifetime and spectral emission contrast between markers and healthy vessel wall is expected, was subsequently imaged with the IVUS/FLIm catheter. In the second animal, a similar procedure was adopted: images were acquired in vessels with diameters ranging from 3 mm to 5 mm using flushing flow rates between 2 cc/s and 8 cc/s to confirm the results from the first animal and evaluate a wider range of vessel sizes.

### *Ex vivo* human coronary imaging

The arteries (n = 21) were provided by the University of Pennsylvania heart transplant program. Whole human hearts were procured from two separate patient groups: non-failing brain dead organ donors with no overt history of heart failure, and end stage heart failure transplant patients. No organs were procured from prisoners. All hearts received *in situ* cold cardioplegia and were placed on wet ice in 4 degree Krebs-Henseleit Buffer. Artery samples were removed and snap frozen in liquid nitrogen and stored at minus 80 °C. Arteries were de-identified prior to shipping from University of Pennsylvania to UC Davis. The study complies with current legal requirements and guidelines and was approved by the University of Pennsylvania Hospital Institutional Review Board as well as UC Davis Biological Use Authorization. Prospective informed consent for research use of heart tissue was obtained from all transplant recipients and next-of-kin in the case of organ donors.

Samples were mounted on a dedicated holder and immersed in 37 °C Phosphate buffered saline (PBS) solution. PBS was circulated inside the vessel during imaging to maintain lumen shape. Following imaging, the samples were fixed under perfusion, and sent to the Texas Heart Institute for further processing, sectioning and staining. Each artery segment was first cut into rings (~2 mm length). Sections were cut at four different locations for each ring, leading to at least 32 histology sections per sample. Visualization of plaque morphology and composition (collagen, elastin, and lipids) was enabled by H&E and Movat’s pentachrome, correspondingly. Immunohistostaining with CD68 was also performed to visualize macrophage content in the vessel wall.

## Electronic supplementary material

Supplemental material
