# Supplementary material for: In vivo label-free structural and biochemical imaging of coronary arteries using an integrated ultrasound and multispectral fluorescence lifetime catheter system
Source: Sci Rep. 2017 Aug 21;7:8960. doi: 10.1038/s41598-017-08056-0 (PMC5566546; doi:10.1038/s41598-017-08056-0)
Supplement: Supplementary file 1 — Supplemental material [file 41598_2017_8056_MOESM1_ESM.doc]

*In vivo* label-free structural and biochemical imaging of coronary arteries using an integrated ultrasound and multispectral fluorescence lifetime catheter system

Julien BEC, M.S., Department of Biomedical Engineering, University of California, Davis, CA

Jennifer E. PHIPPS, PhD, Department of Biomedical Engineering, University of California, Davis, CA

Dimitris GORPAS, PhD, Department of Biomedical Engineering, University of California, Davis, CA

Dinglong MA, Department of Biomedical Engineering, University of California, Davis, CA

Hussain FATAKDAWALA, PhD, Department of Biomedical Engineering, University of California, Davis, CA

Kenneth B. MARGULIES, Cardiovascular Institute, Perelman School of Medicine, University of Pennsylvania, Philadelphia, PA

Jeffrey SOUTHARD, MD, Medical Center, Division of Cardiovascular Medicine, University of California Davis, Sacramento, CA

Laura MARCU, PhD, Department of Biomedical Engineering, University of California, Davis, CA

Department of Biomedical Engineering
University of California, Davis
451 East Health Sciences Drive
Davis, CA 95616

[jbec@ucdavis.edu](mailto:jbec@ucdavis.edu)

# Supplemental material

# Supplemental methods

## Integrated FLIm/ IVUS system

The FLIm/IVUS intravascular imaging system was based on an existing clinical IVUS system (iLab platform with Opticross motor drive and catheter, Boston Scientific). The clinical IVUS system consists of a 40 MHz 3 F catheter connected to a motor drive unit (MDU). The MDU is connected to an acquisition computer. A touchpad console and screen enable user control and real time data display.

Several modifications of the Opticross MDU were required to provide an optical channel to make it compatible with fluorescence imaging. The driving motor was offset using a gear train to provide space for a custom fiber optic rotary joint (FORJ) in line with the rotary shaft. The fiber from the rotating side of the FORJ (200 µm core UV grade multimode fiber optic) was then routed through the radio frequency rotary joint and into the hub where an optical connector based on a spring loaded zirconia ferrule was installed to provide an optical interface with the catheter. The fixed part of the FORJ consisted of a patch cord providing connection to the FLIm system. Optical matching gel (0607, Cargille Laboratories, NJ) was applied both inside of the rotary joint and in the hub to improve transmission.


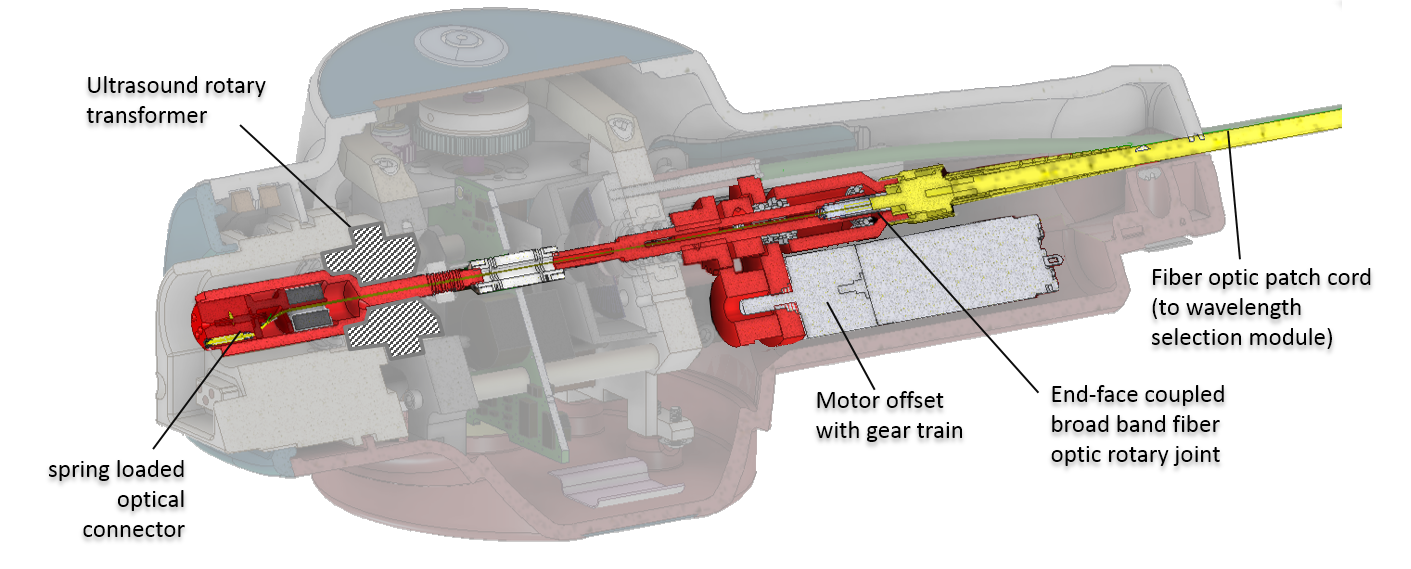


***Supplemental Figure 1: Modified iLab motor drive.*** *Custom parts in red. The motor providing rotation of the catheter was offset using a train of gears to enable integration of a custom fiber optic rotary joint at the back of the shaft. A spring loaded optical connector was integrated into the hub to provide a reliable and repeatable optical coupling with the catheter.*

The FLIm system, described previously^17^, is composed of a pulsed UV fiber laser (20 KHz, 80 ps pulses, Fianium, UK), a wavelength selection module that couples the laser light to the MDU and collects the fluorescence signal and splits it into four different bands tailored to match the emission spectra of biological compounds expected in healthy and diseased arteries:

| Channel number | 1 | 2 | 3 | 4 |
| --- | --- | --- | --- | --- |
| Central wavelength (nm) | 390 | 452 | 542 | 629 |
| Channel width (nm) | 40 | 45 | 50 | 53 |
| Fluorophores (lifetime in ns) | Collagen (6 ns)  Elastin (4.5 ns) Lipoproteins (4 ns)  Cholesteryl oleate (4.5 ns) | Elastin (4.5 ns)  Collagen (6 ns)  Lipoproteins (5 ns) | Elastin (5 ns)  Ceroid  lipofuscin | Ceroid  lipofuscin |

Table S1

The signal from each band is then routed through optical delay lines of different lengths (1, 10, 19 and 28 m) equivalent to a 45 ns delay between each channel. This delay enables emitted fluorescence to be spectrally resolved into the four spectral bands and sent to a single fast multi-channel plate photomultiplier (R3809U-50, Hamamatsu, Japan). The electrical signal is then amplified (C5594 Hamamatsu, Japan) and sampled by a high-speed digitizer (12.5 GS/s, 3 GHz, 8-bit, 512 Mbytes, PXIe-5185, National Instruments, Austin, Texas). This detection scheme enables simultaneous acquisition of fluorescence decay from all four wavelength bands with a single photodetector from each excitation pulse, thus allowing high speed acquisition when compared to alternative fluorescence lifetime methods^17^.

FLIm data were analyzed using a Laguerre basis expansion technique^18^: For each channel, the acquired waveform was fitted on to a set of Laguerre functions convolved with the instrument response function. The fluorescence decay is recovered by performing a sum of the Laguerre basis functions weighted by the coefficients identified in the previous step. Both the average fluorescence lifetime and intensity imaging values were computed from each measurement and maps were obtained where the location of each pixel corresponds to a distinct angular position at a specific pullback location of the catheter. In addition, IVUS data were processed to extract the vessel lumen ^19^ and to map the distance between catheter tip and vessel wall. This distance map was then used to correct the FLIm intensity in *en face* images in each channel caused by variations of light excitation/collection efficiency with probe-to-wall distance ^20^; improving the ability to quantify the amount of fluorescence signal emitted in each location for a given channel. Intensity ratio maps for each channel were created by normalizing the per pixel intensity for a given channel by the sum of intensities of the same pixel from all four channels. Intensity and lifetime information were then combined to create intensity-weighted lifetime maps where the color represents the lifetime value while the brightness represents the absolute amount of fluorescence. This representation is useful to include structures that can be identified based on intensity, such as stent struts shadows. Intensity ratio and lifetime information were also combined to create spectral ratio weighted lifetime maps, where the color represents the lifetime value while the brightness represents the share of total detected fluorescence originating from this specific channel. This representation enables both time resolved and spectral information to be included in the same graphic representation and is independent from variations in excitation/collection efficiency due to distance and geometry. It is therefore a true representation of the intrinsic fluorescence properties of the vessel wall at this location. Finally, fluorescence lifetime data were projected onto the vessel surface derived from IVUS to create 3D fluorescence lifetime maps.


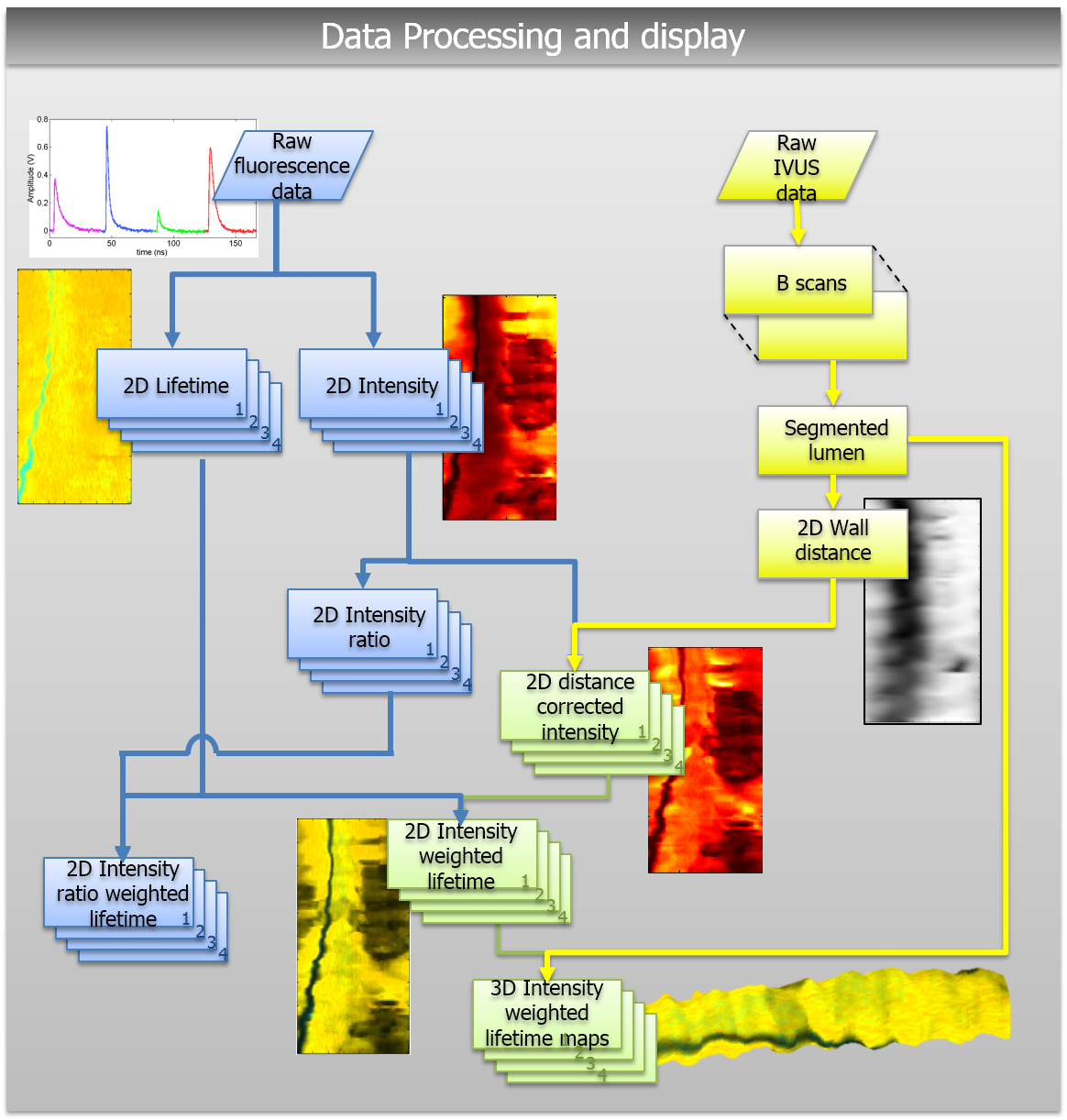


***Supplemental Figure 2: Data processing flow showing how FLIm and IVUS data are combined to provide information.*** *An example of the image is displayed next to each box. The functionality of FLIm is improved by IVUS: fluorescence intensity images is corrected based on probe to vessel wall distance and the FLIm data are displayed in 3D using vessel geometry recovered from IVUS.*

The robustness of lifetime information was evaluated by extracting intensity and lifetime values from the vessel wall for channel 2. (Figures 3a-b). The average lifetime (solid line, red) as well as standard deviation (shaded area, red) was computed and plotted as a function of signal intensity (Figure 3c) and shows minimal variation of lifetime and low standard deviation (<0.27 ns) with respect to intensity. A second pullback acquired in the same location (green) shows similar values. The same procedure applied over 5 pullbacks acquired in a second location (Figure 3 d) resulted in similar results.

## Integrated FLIm/IVUS System: Technical characterization in phantom

The ability of the system to acquire co-registered FLIm and IVUS data was tested in a 3 mm inner diameter fluorescent acrylic tubular phantom (6954 Fluorescent Amber, Polyone, MI), in which a stent was placed to provide contrast. The IVUS images were processed using a segmentation algorithm previously developed by our group^19^. The stent was isolated from the tubular phantom lumen and a skeleton image based on the *en face* maximum intensity projection was created. This image was then compared with the skeleton derived from the *en face* fluorescence intensity image (Figure S3).

## Fluorescence properties of pure lipids and cholesterols

Fluorescence properties of biological compounds depend from the excitation wavelength> To provide reference values, the fluorescence lifetime and emission spectra of compounds expected to be present in diseased arteries, such as lipoprotein, cholesterol and cholesterol esters, as well as elastin and collagen were characterized using identical (355 nm) excitation wavelength as used for ex vivo and in vivo artery measurements. The compounds were sourced (Sigma-Aldrich, St Louis, MO) and tested using a spectrometer based fluorescence lifetime measurement system. The tests were performed using the same fiber laser/ photomultiplier/ preamplifier/ digitizer used in combination with the catheter system but light was dispersed by a spectrometer (MicroHR, Horiba Jobin Yvon, Edison, NJ) instead of the wavelength selection module described above. Measurements were performed in triplicate, at wavelengths between 360 nm and 550 nm with a 5 nm steps. Fluorescence intensity and lifetime were estimated using a similar method to the fluorescence data obtained from the catheter system, and fluorescence and lifetime emission spectra were derived.

## *Ex vivo* coronary imaging

The coronary human samples, obtained from the University of Pennsylvania heart transplant program, were chilled in isopentane, frozen in liquid nitrogen and stored at -80 °C. Before imaging, each sample was thawed. The sample was kept in 37 °C phosphate buffered saline during the whole imaging procedure. After imaging, the specimen, still mounted on the holder, was fixed in 10% buffered formalin. It was then decalcified and 2 mm cuts were performed using the reference guides from the holder to provide axial registration between histology and imaging data. 4 µm thick sections of each cut sample were then stained with hematoxylin and eosin (H&E), and Masson’s trichrome with elastin to visualize plaque morphology and composition (collagen, elastin, and lipid). Immunohistochemical staining with CD68 was also performed to visualize macrophage content in plaque.

## *In vivo* coronary imaging

## In vivo testing of the system was carried out in porcine animal model, in compliance with the UC Davis Institutional Animal Care and Use Committee. The animal was anesthetized and coronary access was achieved using a 9 Fr sheath placed in the femoral artery using a modified Seldinger technique and a 7 Fr hockey stick guide.

# Supplemental results

## Spatial resolution and co-registration performances

Excellent registration was obtained in phantoms, as demonstrated in Figure s3. The overlay of the stent skeletons extracted from each modality demonstrates that a registration error of one pixel or less is typically achieved, corresponding to 140 µm in the axial and 2.2 degrees in the trans-axial direction. Spatial resolution of FLIm, defined as the full width at half maximum (FWHM) of a sub-resolution fluorescence target, was measured to be 180 µm at 2 mm distance from the probe.


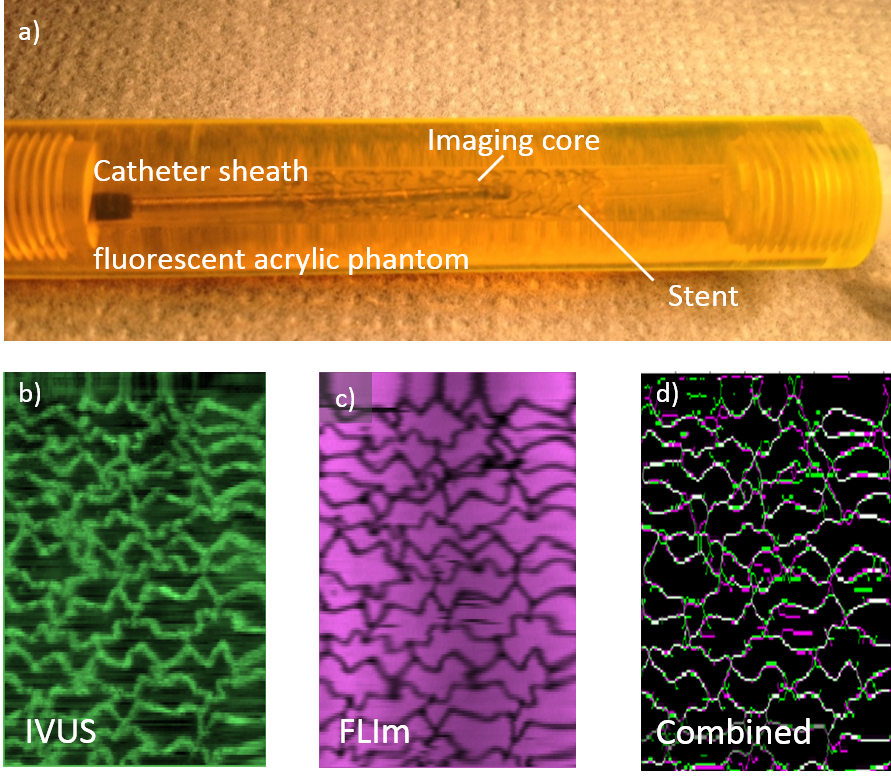


***Supplemental Figure 3: Phantom validation of data co-registration.*** *multimodal catheter inserted in the stented section of a 3mm inner diameter fluorescent acrylic phantom (a). En face image of the stent extracted from the IVUS image (b) and shadow of the stent as seen by FLIm (c) have been processed: the skeletons extracted from each image have been overlaid and show accurate registration.*

## Fluorescence properties of pure lipids and cholesterols

Emission spectra of the tested species are presented in Figure S4. Their emission spectra are very broad and overlapping as demonstrated in the left pane. Focusing on the lifetime spectra, all compounds present a lifetime in the 3.5 to 6 ns range. Pure compounds can be differentiated using both spectral and temporal information based on the standard deviation obtained between measurements, at the exception of HDL and LDL that present virtually identical fluorescence signature.


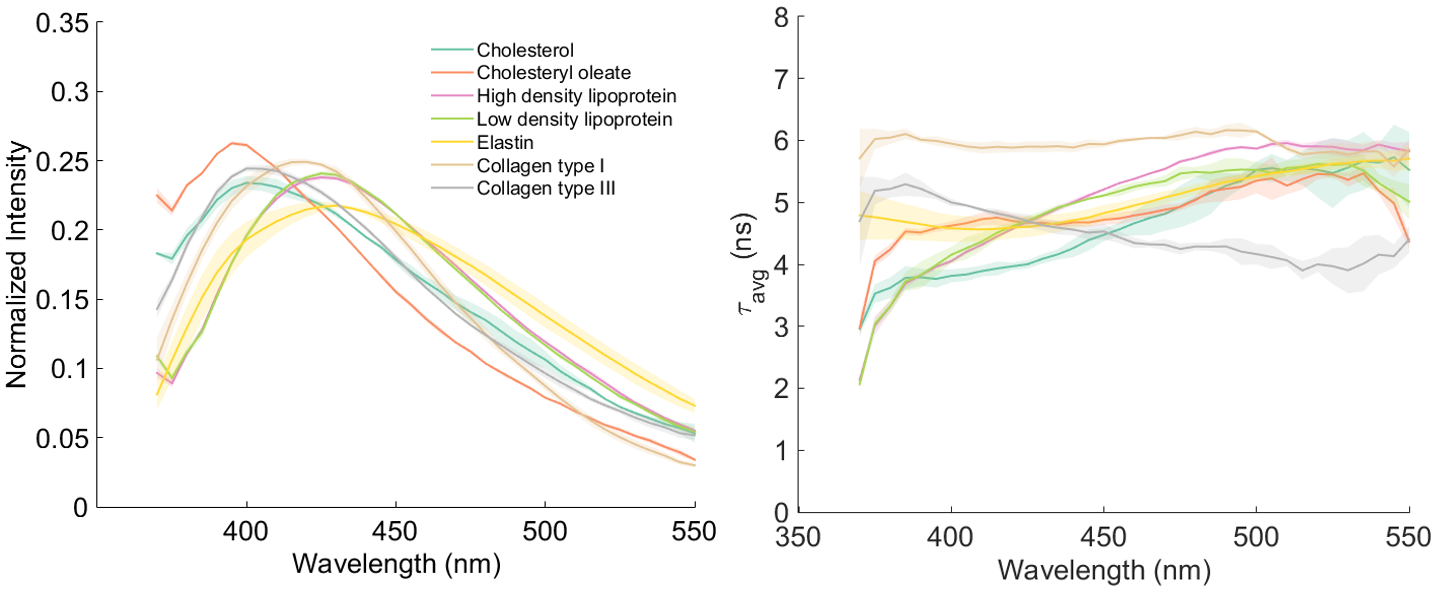


***Supplemental Figure 4: Emission and lifetime spectra of expected compounds.*** *Mean value (solid line) and standard deviation (shaded background) of measured Intensity and lifetime spectra of biological compounds present in arteries. Although these species have significantly overlapping spectra, a combination of spectral and temporal information enables differentiation between species, at the exception of HDL and LDL that present very close signatures.*

## *In vivo* FLIm/IVUS imaging of healthy coronary

The robustness of lifetime information was evaluated by extracting intensity and lifetime values from the vessel wall for channel 2. (Figures S5a-b). The average lifetime (solid line, red) as well as standard deviation (shaded area, red) was computed and plotted as a function of signal intensity (Figure S5c) and shows minimal variation of lifetime and low standard deviation (<0.27 ns) with respect to intensity. A second pullback acquired in the same location (green) shows similar values. The same procedure applied over 5 pullbacks acquired in a second location (Figure S5d) resulted in similar results.


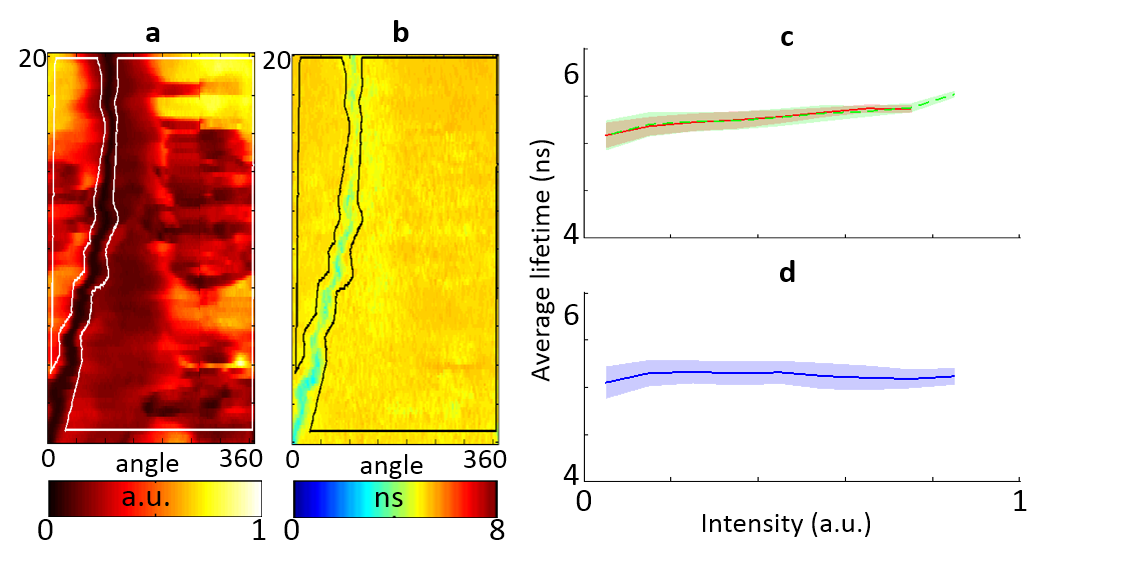


***Supplemental Figure 5: Assessment of the robustness of lifetime information acquired in vivo.*** *Fluorescence intensity (a) and lifetime (b) images have been segmented to remove the guide wire. Lifetime values and standard deviation have then been plotted as a function of signal intensity, for two acquisitions at the same location (c). Lifetime values and standard deviation as a function of signal intensity across five different additional locations (d). Lifetime shows minimal variations with intensity and limited increase in standard deviation as the signal intensity decreases.*

## *Ex vivo* plaque characterization using FLIm/IVUS data

Multispectral FLIm allows clear identification of fibrotic plaque areas, characterized by an increase in collagen content leading to a red-shift of the emitted fluorescence as well as an increase in lifetime in the first channels (Figure S6). The location of the fibrotic plaque areas identified by FLIm matches the location of fibrotic plaque identified both by IVUS and histology (Figure S7). The foam cells infiltration in the superficial layers of the intima at 9 o’clock visible in histology corresponds to an increase in channel 3 emission as well as larger lifetime (>6 ns).


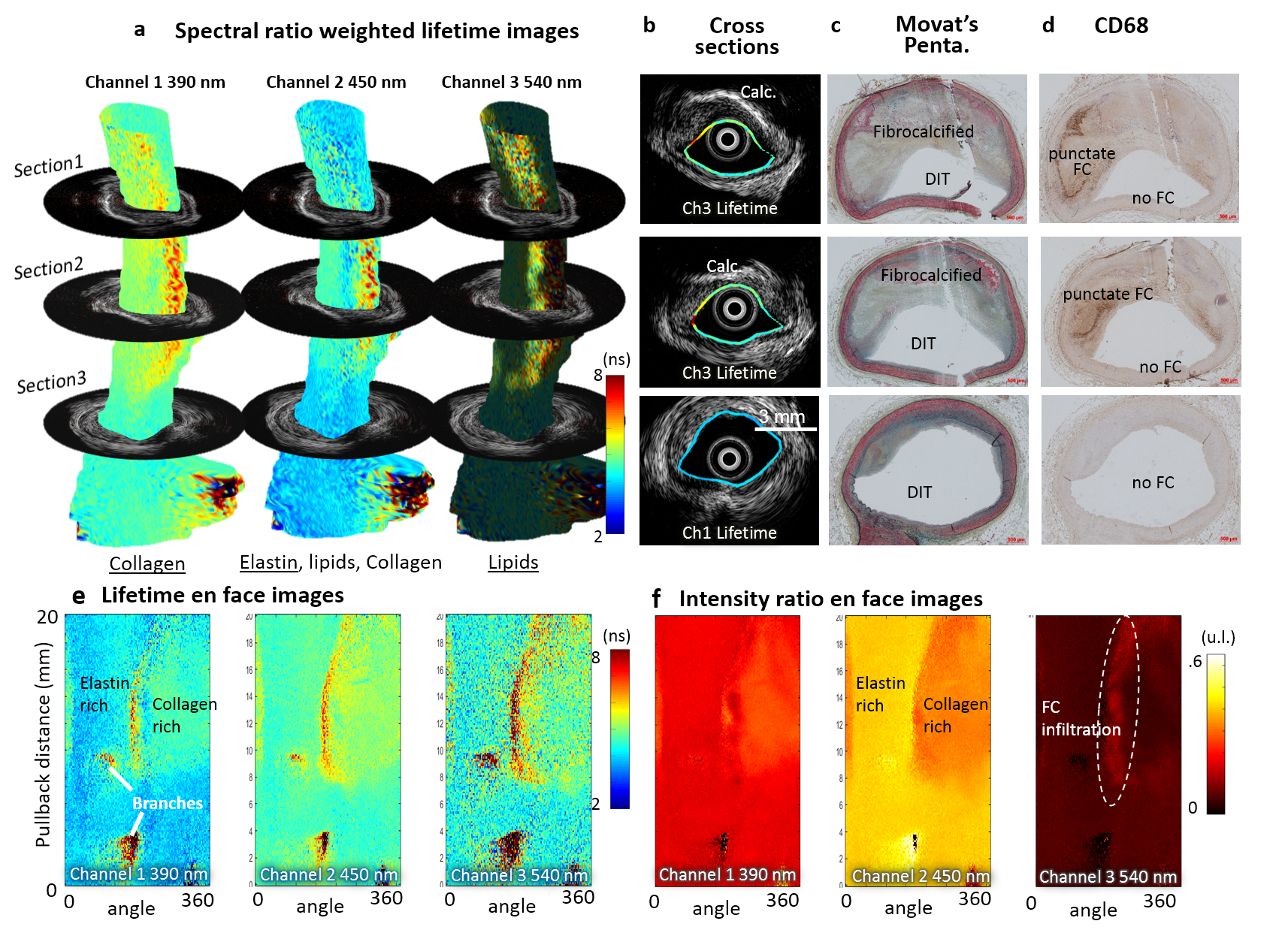


***Supplemental Figure 6****:* ***FLIm biochemical information supplements IVUS for the assessment of atherosclerotic lesion pathophysiology. Spectral ratio weighted lifetime images*** *map the measured lifetime (color), as well as relative strength of the signal (brightness) for channels 1,2 and 3 of the instrument. Collagen is the main contributor in channel 1 fluorescence emission; Elastin and to a lesser extent collagen and some lipids fluoresce in channel 2, whereas only lipids (including ceroid and lipofuscine) present significant emission in channel 3. For 3 sections in the vessel, IVUS cross sections with fluorescence overlays (b) were matched with Movat’s pentachrome (c) and CD68 (d) stained sections.* ***Section 1*** *presents a fibrocalcified lesion with foam cells infiltration at 9 o’ clock (see CD68). Fluorescence emission from foam cells is identified in channel 3 and presents longer lifetime than surrounding areas. DIT and fibrotic regions differ by the ratio of elastin to collagen: fibrotic regions present longer channel 1 lifetime and a red-shifted emission.* ***Section 2*** *corresponds to a second section of the same lesion and presents similar characteristics.* ***Section 3*** *presents a DIT region whith homogeneous fluorescence signature dominated by elastin emission.* ***En face images*** *of* ***lifetime*** *(e) and* ***intensity ratio*** *(f) used to compute ratio weighted lifetime images shown in (a)*
